# Increasing TB/HIV Case Notification through an Active Case-Finding Approach among Rural and Mining Communities in Northwest Tanzania

**DOI:** 10.1155/2022/4716151

**Published:** 2022-03-12

**Authors:** R. Abeid, C. Mergenthaler, V. Muzuka, A. Goodluck, T. Nkwabi, J. Bigio, Aguilera Vasquez N, T. Pande, F. Haraka, J. Creswell, T. Rahman, M. Straetemans

**Affiliations:** ^1^SHDEPHA+ Kahama, Shinyanga, Tanzania; ^2^KIT Royal Tropical Institute, Amsterdam, Netherlands; ^3^Research Institute of the McGill University Health Centre, Montreal, Canada; ^4^Ifakara Health Institute, TB Interventions and Clinical Trials Department, Ifakara, Tanzania; ^5^Elizabeth Glaser Pedatric AIDS Foundation, Dar esa Salaam, Tanzania; ^6^Innovations & Grants Team, Stop TB Partnership, Geneva, Switzerland

## Abstract

While Tanzania is among the high TB burden countries to reach the WHO's End TB 2030 milestones, 41% of the people estimated to have had TB in 2020 were not diagnosed and notified. As part of the response to close the TB treatment coverage gap, SHDEPHA+ Kahama conducted a TB REACH active case-finding (ACF) intervention among rural and mining communities in Northwest Tanzania to increase TB/HIV case notification from July 2017 to June 2020. The intervention successfully linked marginalized mining communities with integrated TB/HIV screening, diagnostic, and referral services, screening 144,707 people for TB of whom 24,200 were tested for TB and 4,478 were tested for HIV, diagnosing 1,499 people with TB and 1,273 people with HIV (including at least 154 people with TB/HIV coinfection). The intervention revealed that community-based ACF can ensure high rates of linkage to care among hard-to-reach populations for TB. Providing integrated TB and HIV screening and diagnostic services during evening hours (Moonlight Events) in and around mining settlements can yield a large number of people with undiagnosed TB and HIV. For TB, this is true not only amongst miners but also FSW living in the same communities, who appear to be at similar or equally high risk of infection. Local NGOs can help to bridge the TB treatment coverage gap and to improve TB and HIV health outcomes by linking these marginalized groups with public sector services. Capturing the number of referrals arriving at CTCs is an important next step to identify how well the integrated TB/HIV outreach services operate and how they can be strengthened.

## 1. Introduction

Diagnosis and treatment of *tuberculosis* (TB) are vital components of the End TB Strategy target to reduce the number of TB deaths by 95% by 2035 from 2015 [1]. Treatment coverage, an estimate of the proportion of people with TB who are detected and notified, is therefore an important measure of access to TB diagnosis and care [2]. The World Health Organization (WHO) estimated Tanzania's 2020 *tuberculosis* (TB) treatment coverage cases to be 64%, which has been improving as estimated incidence decreases despite modest changes in the total number of people notified [3]. This estimate implies that 41% of people with TB in 2020 were not diagnosed and notified in Tanzania, contributing to excess mortality [4].

Tanzania's TB incidence rate is estimated at 222 per 100,000 population (0.22%), and its HIV prevalence rate is estimated at 4.8% (95% CI: 4.1–5.3%); as of 2019, HIV coinfection among people with TB was 24% [3,5]. Tanzania is designated as one of WHO's 30 high burden countries for TB and TB/HIV, and despite its 2019 population of 58 million people, 5% of all people globally with TB/HIV live in the country [2].

While TB treatment coverage in Tanzania is below global averages, within the country there are also important differences. The London School of Health and Tropical Medicine's subnational burden estimation tool SUBSeT places regions in northwestern Tanzania among those with the lowest treatment coverage with various areas under 40% [6]. One of the potential reasons for low treatment coverage is the presence of several key populations for TB who tend to suffer from poor access to health services, stigma, and discrimination [7, 8]. A large population of artisanal small-scale miners (ASMs) resides in northwest regions, such as Shinyanga and Geita. ASMs often live in informal settlements surrounding the mines and have limited access to healthcare services [9, 10]. Through their work, ASMs are exposed to numerous toxic substances including silica dust, which can cause silicosis, a risk factor for developing TB [11]. A recent study showed that non-HIV infectious diseases (respiratory infections including *tuberculosis*) were the leading cause of mortality in two mines in Geita and Shinyanga provinces [12]. Many female sex workers (FSWs) also live in and around the same informal settlements. Rates of human immunodeficiency virus (HIV) infection are high among both ASMs and FSWs, increasing their risk of developing TB [10]. However, despite the presence of risk factors for TB, the TB prevalence among both risk groups remains unexplored.

Geographical and financial factors limit access to TB and HIV health services in Shinyanga and Geita regions. The areas are predominantly rural, connected by a poor road network, and have only an estimated 1.1 health facilities per 100,000 population compared to the national average of 1.5 per 100,000 [13]. In addition, 37.3% and 19.1% of Shinyanga and Geita's respective populations fall into the lowest wealth quintile [14, 15].

Due to the limitations of the public health system and large numbers of key populations in the region, community-based organizations play a critical role in supporting TB and HIV health services to provide outreach and care. With Stop TB Partnership's TB REACH funding, the community-based NGO SHDEPHA+ Kahama conducted a TB active case-finding (ACF) intervention evaluation in Shinyanga and Geita regions in an attempt to answer two questions: (1) to what extent did the integrated TB/HIV ACF intervention link presumptive for TB to TB and HIV testing and treatment services? and (2) what is the estimated prevalence of TB among ASM and FSW?

## 2. Methods

### 2.1. TB Active Case-Finding and Integrated HIV Testing Intervention

#### 2.1.1. Setting and Population

**Figure 1:**
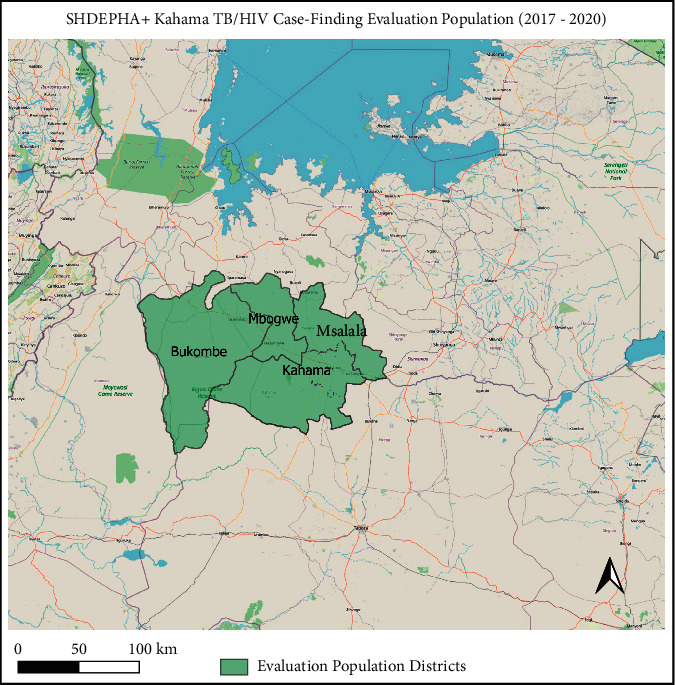
TB/HIV case-finding intervention area (2017–2020).

As part of its TB REACH intervention, SHDEPHA+Kahama conducted TB ACF in mining communities and other hard-to-reach areas of Kahama and Msalala districts of Shinyanga region from July 2017 until September 2018. The intervention expanded to Mbogwe and Bukombe districts of Geita region in October 2018 and lasted through June 2020 (Figure 1). Four population categories were defined as follows: hard-to-reach community adults age 15 and above (nonmining communities), children age ≤15 (mining and nonmining communities), adult male and female miners living in mining communities ≥15 years of age, and FSWs age 18 and above. The latter two were the predominant groups screened in mining settlements. FSWs were identified primarily by using a screening tool adapted from PEPFAR guidelines in mining communities and partially in brothels and bars in the vicinity [16]. While a control population was monitored for the larger TB REACH intervention, for the specific research questions addressed in this manuscript, a control was not prioritized.

#### 2.1.2. TB and HIV Screening

**Figure 2:**
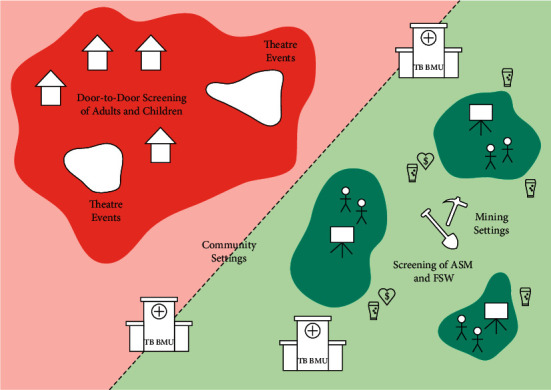
Types of active case-finding interventions conducted in Shinyanga and Geita regions, Tanzania.

The intervention focused on TB ACF, and in January 2019, expanded to include HIV testing and referral to care and treatment centers (CTCs). ACF was conducted in community and ASM settings: in communities, SHDEPHA+Kahama conducted door-to-door screening and theatrical TB sensitization events, and in ASM settings, large TB awareness and screening events at the mines and nearby bars and brothels. During door-to-door screenings, peer educators (PEs) provided one-on-one education about TB symptoms and risk factors; actors explained these during theatrical events when they were arranged in selected communities. In mining settlements, community members were invited to a “moonlight-screening event” at a central location during evening hours to view educational videos on TB/HIV information on a large projected screen, hosted by a staff member who would also moderate a question-and-answer session (Figure 2).

For all ACF activities, peer educators (PEs) implemented a verbal screening for TB symptoms including cough of any duration, weight loss, fever, excessive night sweats, history of living with a TB patient by using community TB/leprosy-screening questionnaires (TSQs). Any one of the National TB Program's five symptoms triggered a presumptive status as per national guidelines, followed by sputum collection by a community health worker (CHW) or PE [17]. During verbal TB screening, individuals were also asked about their HIV status and offered a rapid HIV test. Regardless of the presence of TB symptoms, an HIV-positive or unknown HIV status triggered CHWs and PEs to refer individuals to the nearest HIV care and treatment center (CTC), and referred individuals were given a lab referral form to be presented to the CTC for testing or treatment services.

#### 2.1.3. Diagnosis and Treatment

TB specimens were transported to the nearest TB basic management unit (BMU) laboratory for testing with Xpert MTB/RIF (Xpert) when possible; otherwise, light microscopy was performed. When bacteriological testing was not possible, clinical evaluation was performed. A positive test on either Xpert or smear microscopy was considered bacteriologically positive (*B*+) for TB, and individuals diagnosed with TB were enrolled on treatment. CHWs provided treatment support by delivering anti-TB drugs to all patients identified by the ACF intervention. When referred individuals arrived at a CTC, HIV diagnosis and care were provided according to National AIDS Control Programme Guidelines for collaborative TB/HIV activities [18]. For individuals in whom HIV was diagnosed but not TB, isoniazid preventive treatment (IPT) was started.

### 2.2. Data Collection

SHDEPHA+Kahama developed paper-based data collection tools to collect data along the TB care cascade for the ACF intervention. These tools also captured demographic data and population category (general adult population, children, ASMs, and FSWs). Additionally, BMUs in the evaluation population districts coordinated with CTCs to assemble data on the HIV status of TB patients referred for HIV diagnosis or care by SHDEPHA+Kahama. Thus, the field team collected data from the nearest engaged BMUs to identify gaps and drop-offs in HIV care and to merge CTC with TB data.

Field staff also sent aggregate intervention data (e.g., number screened and number identified as presumptive for TB) via SMS to project officers on a weekly basis, which were cross-checked with aggregate data collected on paper. Data were digitally entered using Epidata Entry (version 3.1), reviewed on a monthly and quarterly basis between October 2018 and March 2020 in Epidata Analysis (version 2.2.3.187).

### 2.3. Data Analysis

TB-screening data generated during ACF activities, laboratory data from facilities where specimens were tested, and CTC data were transferred from EpiData to Stata version 15.0 for final analysis. The datasets were merged on the district, quarter, population category, and gender. Proportions of individuals passing to each stage of the screening cascade (screening, diagnosis, and treatment initiation) were calculated. Descriptive statistics for TB and HIV pathway of care indicators were calculated across population categories and overall. To estimate TB prevalence in each population group, we divided the number of people diagnosed with TB by the number of individuals verbally screened for TB. Chi-square tests were run to test for differences in passage to subsequent steps in the cascade by population category. When significant differences (*p* < 0.05) were identified from the chi-square tests, proportions tests were computed comparing all population groups against each other, per cascade indicator. We applied the Bonferroni correction and divided the alpha level of 0.05 by the number of group comparisons (6: adults vs children, adults vs FSW, adults vs ASM, children vs FSW, children vs ASM, and FSW vs ASM) to minimize the Type I error rate. As a result, we identified a revised alpha of 0.008 required to compare multiple proportions.

## 3. Results

### 3.1. TB Screening and Diagnosis

A total of 144,707 individuals were screened for TB in the communities and ASM settlements (Table 1). Overall, 55,461 (38.3%) were identified as presumptive for TB based on verbal symptoms, of whom 24,200 (43.6%) were tested for TB by either microscopy or GeneXpert. Of those tested, 1,499 (6.2%) had a bacteriologically positive TB diagnosis, which corresponds to an overall prevalence among people screened of 1.0%. Chi-square tests revealed statistically significant differences at all stages of the TB and HIV care cascade, and proportions tests revealed significant differences in all population group comparisons for presumptives among screened, presumptives among tested, and *B*+ cases among tested (Table 2). Adults in hard-to-reach communities and FSWs had higher proportions of presumptive individuals (39.5% and 45.6%, respectively) compared to other populations screened, while children and ASM had a higher proportion of bacteriologically tested or clinically evaluated (53.2% and 49.6%, respectively) (Table 1). FSWs and ASM had higher rates of *B*+ TB among those tested (8.3% and 10.0%, respectively), and ASM had the highest treatment initiation rates (16.6%). *B*+ and AF treatment initiation rates showed no significant differences between groups.

### 3.2. HIV Screening and Diagnosis

Overall, 4,478 (18.5%) individuals who were tested for TB were also tested for HIV either in the communities, at moonlight events, or a CTC, of whom, 1,273 (28.4%) were HIV positive. Adults had the highest likelihood of being tested for HIV (21.3%), and FSWs were most likely to have an HIV-positive diagnosis (50.3%) (Table 3). In total, 154 individuals were diagnosed with HIV and all forms of TB. A total of 1,159 people with HIV who were bacteriologically negative for TB were started on isoniazid prophylaxis treatment (IPT). Proportion tests showed that adults with TB were significantly more likely than the other groups (with TB) to be tested for HIV (Table 2).

Amongst individuals verbally screened for TB, ASM and FSW had similar estimated *B*+ TB prevalence rates at 1.8% and 1.7%, respectively and 3.0% for all forms of TB prevalence (Table 4). Estimated *B*+ TB prevalence among verbally screened adults and children in communities were 0.9% and 0.6%, respectively, and all forms TB prevalence among verbally screened adults and children in communities were 1.8% and 2.1%, respectively. The number needed to screen (NNS) to find one person with *B*+ TB was lowest in ASM (56) and FSW (60), as presented in Table 4.

## 4. Discussion

The intervention successfully linked marginalized mining communities with integrated TB/HIV-screening, diagnostic, and referral services, testing 144,707 people for TB, of whom 24,200 were tested for TB and 4,478 were tested for HIV, and diagnosing 1,499 people with TB and 1,273 people with HIV (including at least 154 people with TB/HIV coinfection). We found significant differences between nearly all population groups advancing through the care cascade, except for TB treatment initiation. We also showed that community-based ACF can ensure high rates of linkage to care among hard-to-reach populations for TB, while numerous studies have shown that even in routine case finding, there can be high rates of pretreatment loss to follow up for both TB and HIV [19, 20].

Our findings document very high rates of TB and HIV in hard-to-reach communities and ASM communities. *B*+ TB prevalence estimates were similar between ASMs and FSWs (1.8% and 1.7%, respectively), twice as high as the rates measured in adults and children of hard-to-reach communities in our study setting (0.9% and 0.6%, respectively), and therefore up to eight times as high as Tanzania's general population incidence rate [3].

### 4.1. TB/HIV Case-Finding Cascade

Only 43.6% of people with presumptive TB were tested for TB in spite of on-the-spot specimen collection. In our setting, no travel was required to access specimen collection, as project staff collected sputum samples at the point of verbal screening; however, we could not capture reasons for refusal. Other ACF interventions have also had low rates of sputum collection [21, 22]. In an ACF intervention in Bihar, India, 44% of 11,146 people with presumptive TB were tested; however, presumptive TB patients were accompanied by a CHW to a testing point. Barriers identified in the Bihar study included TB stigma, insufficient transport, insufficient support of families and providers, and poor public health services, while other ACF studies cited reasons including difficulty producing sputum, light symptoms, transport challenges, insufficient family and provider support, and perceived poor services in the public sector [23, 24].

Because we were not able to collect sputum and test a large proportion of people with a positive screen, it is likely that we missed a potentially large number of people with TB; in addition, several studies and prevalence surveys have documented that a large proportion of people with *B*+ TB do not report symptoms and are only identified by chest X-ray [25–27]. Limited evidence exists around strategies to link people attending TB ACF events to HIV services [28]. However, even in facility settings, TB patients are reluctant to receive HIV counseling and testing, and individuals diagnosed with HIV in mobile settings show modest antiretroviral treatment initiation rates [29, 30]. Our study showed a large drop-off in the link to HIV testing amongst those tested for TB, with HIV high-risk groups like ASM and FSW even less likely to appear for HIV testing. Reasons for this should be explored to understand what additional support is needed in the way of transportation, financial, or psychosocial to minimize the loss of people who should be linked to care.

### 4.2. TB Prevalence

Widely known risk factors for TB and HIV place populations living in mining communities at a higher risk for TB and/or HIV (co)infection [10, 31, 32]. As expected, more ASM and FSW who were tested for TB received bacteriologically positive TB diagnoses (8.3% and 10.0%, respectively) compared to the adults and children in hard-to-reach communities. Among the 1,499 new and relapse *B*+ TB cases detected, 505 (34%) were found in and around mining settlements, which made up only 20.5% of all people screened, illustrating an undue burden of TB. When applied to the underlying populations in attendance of the screening events, ASM and FSW had similar rates of new and relapse *B*+ TB and all forms TB between 1.8 and 2.1% for new and relapse *B*+ and 3.0% for all forms TB. This is ten times the estimated all forms TB incidence rate of 237 per 100,000 (95% CI: 112–408), or approximately 0.2% [3]. Although it is well documented that miners are at increased risk for TB, our study provides an estimated prevalence of TB in FSWs living in and around mining settlements. Other studies have produced gender-disaggregated results of TB screening and diagnosis in mining settlements but documented lower risk for TB in females [33] or similar burden for female miners only [34]. Although a study examining TB and HIV prevalence, knowledge, and behavior in two mining settlements of Gaza province, Mozambique showed a 0.3% TB prevalence rate and 24.2% HIV prevalence rate among a population of 1,012 adults, gender-disaggregated analysis was not presented [31]. Based on our study's overlapping 95% confidence intervals for *B*+ TB prevalence rates and the same all forms TB prevalence estimate (3.0%), these findings suggest that burden among FSWs, who are not directly involved in mining may be similar to miners themselves and more importantly, is incredibly high.

### 4.3. Limitations

The main limitations were challenges related to data collection and management. During screening events, not all people tested for HIV were documented and some people bypassed HIV testing, so the number of people tested for HIV (*n* = 1,273) appears to be an undercount of at least 40 individuals, as evidenced by the fact that TB-/HIV+ IPT initiators and TB/HIV patients total 1,313. Furthermore, HIV status knowledge could not always be captured during the screening events, meaning that those diagnosed with HIV may have known their status beforehand. Bacteriologically confirmed TB was not disaggregated by the type of diagnostic, and the distinction between the number of laboratory tests and clinical evaluations for children was not captured. However, given smear microscopy's lower sensitivity than Xpert, the *B*+ prevalence is certainly an underestimation. Finally, the number of individuals referred to and arriving at CTCs was not captured, which is an important indicator of the ability to link TB and HIV services in outreach settings.

Similar data management challenges are often encountered in operational research settings, and these findings highlight the importance of establishing strict data entry and management protocols, especially when multiple individual-level databases maintained by different individuals must be merged. Clear role divisions should be established including a data quality monitor, and frequent data quality checks should be performed [35]. Tools and guidelines are available for this purpose, and more recently for ACF settings from the Stop TB Partnership [36, 37].

## 5. Conclusion

Providing integrated TB and HIV screening and diagnostic services during evening hours in and around mining settlements can yield a large number of people with undiagnosed TB and HIV. For TB, this is true not only amongst miners but also FSWs living in the same communities, who appear to be at similar or equally high risk of infection. Local NGOs can help to bridge the TB treatment coverage gap and to improve TB and HIV health outcomes by linking these marginalized groups with public sector services. Capturing the number of referrals arriving at CTCs is an important next step to identify how well the integrated TB/HIV outreach services operate and how they can be strengthened.

## Figures and Tables

**Table 1:** TB care cascade for people attending TB-screening events, with population group disaggregated.

	Adults in hard-to-reach communities		Children in hard-to-reach communities		Female sex workers (FSWs)		Artisanal scale miners (ASMs)		Chi-square *p* value	Total	
TB-screening cascade	*n*	%	*n*	%	*n*	%	*n*	%		*n*	%
Individuals screened for TB	100,461		15,663		7,447		21,136			144,707	
Individuals identified with presumptive TB (% among screened for TB)	39,639	39.5	4,756	30.4	3,393	45.6	7,673	36.3	<0.001	55,461	38.3
Individuals tested for TB (% among TB presumptives)	16,364	41.3	2,529	53.2	1,501	44.2	3,806	49.6	<0.001	24,200	43.6
Individuals with bacteriologically confirmed (*B*+) TB (% among tested for TB)	904	5.5	90	3.6	125	8.3	380	10.0	<0.001	1,499	6.2
Individuals with all forms TB (% among tested for TB)	1,829	11.2	325	12.9	225	15.0	630	16.6	<0.001	3,009	12.4
Individuals with *B*+ TB initiated on treatment (% among individuals with *B*+ TB)	869	96.1	90	100.0	125	100.0	371	97.6	0.021	1,455	97.1
Individuals with all forms TB initiated on treatment (% among individuals with all forms TB)	1,788	97.8	324	99.7	225	100.0	622	98.7	0.008	2,959	98.3

**Table 2:** Proportion tests for all cascade indicators, with all population group comparisons.

	Proportional differences between population groups per cascade indicator											
	Adults-children		Adults-FSW		Adults-ASM		Children-FSW		Children-ASM		FSW-ASM	
	Diff.	95% CI	Diff.	95% CI	Diff.	95% CI	Diff.	95% CI	Diff.	95% CI	Diff.	95% CI
% TB presumptive among screened	**0.091**	(0.083, 0.099)	**−0.061**	(−0.073, −0.049)	**0.032**	(0.025− 0.039)	**−0.150**	(−0.165, −0.139)	**−0.059**	(−0.069, −0.049)	**0.093**	(0.080, 0.106)
% TB tested among TB presumptives	**−0.119**	(−0.134, −0.104)	**−0.029**	(−0.046, −.012)	**−0.083**	(−0.095, −0.071)	**0.090**	(0.068, 0.112)	**0.036**	(0.018, 0.054)	**−0.050**	(−0.074, −0.034)
% *B*+ detected among TB tested	**0.019**	(0.011, 0.027)	**−0.028**	(−0.042, −0.014)	**0.045**	(0.040, 0.050)	**−0.050**	(−0.063, −0.031)	**0.026**	(0.018, 0.034)	**0.073**	(0.059, 0.087)
% AF detected among TB tested	−0.017	(−0.031, −0.003)	**−0.038**	(−0.057, −0.019)	**−0.054**	(−0.067, −0.041)	−0.021	(−0.043, 0.001)	**−0.037**	(−0.055, −0.019)	−0.016	(−0.038, −0.006)
% *B*+ on treatment among *B*+ detected	−0.039	(−0.052, −0.026)	−0.039	(−0.052, −0.026)	−0.015	(−0.035, 0.005)			0.024	(0.009, 0.039)	0.024	(0.009, 0.039)
% AF on treatment among AF detected	−0.019	(−0.028, −0.010)	−0.022	(−0.029, −0.015)	−0.009	(−0.020, 0.002)	−0.003	(−0.009, 0.003)	0.01	(−0.001, 0.021)	0.013	(0.004, 0.022)
% tested for HIV among TB tested	**0.094**	(0.080, 0.108)	**0.084**	(0.066, 0.102)	**0.083**	(0.071, 0.095)	−0.010	(−0.031, 0.011)	−0.011	(−0.028, 0.006)	−0.001	(−0.021, 0.019)
% testing HIV+ among HIV tested	**0.143**	(0.010, 0.186)	**−0.210**	(−0.282, −0.138)	0.070	(0.030, 0.110)	**−0.350**	(−0.434, −0.272)	−0.073	(−0.128, −0.018)	**0.280**	(0.200, 0.360)

‡ Proportion test could not run due to comparison of 100% proportion in both groups. *p* < 0.008 based on Bonferroni correction. Bold means chi-square tests statistical significant differences between nearly all population groups advancing through the care cascade, except for TB treatment initiation.

**Table 3:** HIV referral care cascade for people attending TB-screening events, with population group disaggregated.

	Adults in hard-to-reach communities		Children in hard-to-reach communities		Female sex workers (FSWs)		Artisanal scale miners (ASMs)		chi-square *p* value	Total	
HIV-screening cascade	*n*	%	*n*	%	*n*	%	*n*	%		*n*	%
Individuals tested for HIV (% among tested for TB)	3,490	21.3	301	11.9	193	12.9	494	13.0	<0.001	4,478	18.5
Individuals testing positive for HIV (% among tested for HIV)	1,021	29.3	45	15.0	97	50.3	110	22.3	<0.001	1,273	28.4
Individuals started on IPT (who tested bacteriologically negative for TB and HIV+)	802		43		96		218			1,159	
Individuals diagnosed with all forms of TB and HIV	118		5		8		23			154	

**Table 4:** Estimated prevalence of TB, with population group disaggregated.

	Number needed to screen (NNS) to find one person with TB		*B*+ TB prevalence rate		All forms TB prevalence rate	
Key population	*B*+ TB	All forms TB	% Point estimate (95% CI)		% Point estimate (95% CI)	
Adults in hard-to-reach communities	111	55	0.9%	(0.8–1.0%)	1.8%	(1.7–1.9%)
Children in hard-to-reach communities	174	48	0.6%	(0.5–0.7%)	2.1%	(1.9–2.3%)
Female sex workers (FSWs)	60	33	1.7%	(1.4–2.0%)	3.0%	(2.6–3.4%)
Artisanal scale miners (ASMs)	56	34	1.8%	(1.6–2.0%)	3.0%	(2.8–3.2%)
Total	97	48	1.0%	(0.9–1.1%)	2.1%	(2.0–2.2%)

## Data Availability

The data used to support the findings of this study are available upon request to the corresponding author.
